# Observations on the health of infants at a time of rapid societal change: a longitudinal study from birth to fifteen months in Abu Dhabi

**DOI:** 10.1186/s12887-018-1016-z

**Published:** 2018-02-07

**Authors:** Hazel Gardner, Katherine Green, Andrew S. Gardner, Donna Geddes

**Affiliations:** 1 School of Molecular Sciences, University of Western Australia, Crawley, WA 6009 Australia; 2 School of Education, Capella University, 225 South 6th St, Minneapolis, MN 55402 USA

**Keywords:** Infant health, Low birth weight, Developing country, United Arab Emirates, Abu Dhabi

## Abstract

**Background:**

Rapid economic and cultural transition in the United Arab Emirates has been accompanied by a rise in chronic disease. Early childhood is known to affect health outcomes in adulthood. This prospective longitudinal study examined the general health of Emirati infants born in a government maternity hospital in the Emirate of Abu Dhabi in October 2002.

**Methods:**

One hundred twenty-five women, who had recently given birth, were interviewed as part of a larger study encompassing a wide range of cultural, social, and behavioural aspects of health. They were then re-interviewed at three (*n* = 94), six (*n* = 59) and 15 months postpartum (*n* = 52). Data are presented using univariate statistics.

**Results:**

In this study seven infants (6%) were born prematurely and four infants (3%) were classified as small for gestational age, while 11 (9%) of the infants weighed less than 2500 g. Low birth weight infants (LBW) were significantly more likely to require treatment in the neonatal intensive care unit (OR = 30.83, *p* = 0.00). Iron supplementation during pregnancy was associated with fewer underweight infants (OR = 3.92, *p* = 0.042). No associations were found between infant birth weight and maternal age, age at marriage, consanguinity, education level, current maternal employment, parity, pre-existing anaemia or anaemia in pregnancy, diabetes, folic acid intake, multivitamin intake or infant gender.

Maternally-reported infant health issues, vaccination, medication, breast-feeding and infant nutrition, and use of secure car seats are also reported.

**Conclusions:**

The health of infants at birth in this UAE sample showed improvements compared to previous studies. The proportion of LBW infants is decreasing and continuing improvements in health care in the UAE are having a positive impact on infant health.

## Background

The United Arab Emirates (UAE) is a country that is undergoing rapid modernisation yet is experiencing high levels of chronic disease; particularly obesity, heart disease and diabetes [1]. Susceptibility to development of chronic disease is influenced by events occurring in early life [2, 3]. This study explores factors influencing health in infancy in a cohort of 125 Emirati infants.

Globally, in 2015 2.7 million children died in their first 28 days of life resulting in a neonatal mortality rate of approximately 19 per 1000 live births [4]. Almost one million neonatal deaths occurred on the day of birth, and close to 2 million in the first week of life [4]. The main causes of death are pre-term birth complications, intrapartum related complications and neonatal sepsis [5]. The infant mortality rate is an important gauge of development, particularly in relation to socio-economic conditions and provision of health care.

In the postnatal period common causes of death and disability include: pre-maturity (birth before 37 weeks of gestation); neonatal sepsis; respiratory infections; neonatal tetanus; cord infections; congenital anomalies; and birth trauma or asphyxia [6]. In developing countries, infections are still a major cause of death and are preventable by ensuring that births take place under hygienic conditions with trained maternity staff.

In the UAE, it is now mandatory for all women to give birth in hospital with trained staff in attendance, and facilities are on a par with many maternity hospitals in developed countries. Neonatal mortality in the UAE has significantly decreased since 1978 from 17.8 deaths per 1000 births to 3.5 deaths per 1000 births in 2015 [7]. This significant decrease reflects the improvements in living standards and quality of health care in the UAE.

Low birth weight (LBW) infants are most at risk of neonatal death; both preterm infants and those small for gestational age (SGA). In south Asia and sub-Saharan Africa, over 80% of neonatal deaths are of LBW infants [8]. The prevalence of LBW was estimated to be 15% worldwide in 2011 [9]. Defined as weighing less than 2500 g, LBW is the major determinant of morbidity, mortality and disability among neonates and has a long-term effect on health throughout the lifespan. LBW can be a result of preterm birth or intra-uterine growth retardation (IUGR). The highest prevalence of underweight infants is in South Asia and Sub-Saharan Africa [9]. LBW in the UAE had reduced significantly from 15% in 1995 to 6% in 2012 according to UNICEF country estimates [10, 11]. Factors known to impact on birth weight in the UAE include: closely spaced multiple pregnancies which begin at an early age, childbearing into their 40s, high rates of gestational type 2 diabetes during pregnancy, and high prevalence of maternal anaemia. [12]

The mortality rate in Al Ain in 1991 was reported to be 6.7 per 1000 live births, with higher mortality related to lower birth weight [13]. There was a 50% mortality rate in infants with extremely low birth weight (ELBW; less than 1000 g), 20% in very low birth weight infants (VLBW; 1000–1499 g) and 3.1% in moderately low birth weight infants (1500–2499 g). Further, the mortality rate of infants weighing less than 2500 g was 20 times greater than infants weighing above 2500 g [13]. A total of 54 neonatal deaths were reported in the study, 20 from lethal congenital malformations, while 33 were LBW infants, which accounted for 61% of the neonatal deaths. The neonatal mortality rate among UAE nationals in this study was 5.8 per 1000 live births and 6.7% of infants were of LBW [13].

This study examined factors influencing infant health at birth and over the first 15 months of life in a cohort of infants in the city of Abu Dhabi in the UAE in 2002, a time of rapid societal change. More specifically the study focuses on investigating factors contributing to low birth weight and evaluating maternal reported health status of children.

## Methods

This paper focuses on data collected in relation to infant health at birth through to 15 months of age as part of a larger study encompassing a wide range of cultural, social, and behavioural aspects of health in a cohort of women and infants from Abu Dhabi. One hundred and twenty five Emirati women, together with their husbands or guardians, provided written, informed consent to participate in the study, which was approved by the Human Research Ethics Committee at Zayed University, Abu Dhabi, UAE on 12 June 2002. Questionnaires were designed following input from international consultants and Emirati female researchers, who ensured cross-cultural equivalence of the instruments [14]. All materials were created in English and then translated into Arabic using a cross-translation technique [15]. Under this technique an Emirati female research assistant translated the English document into Arabic, and then another Emirati assistant (blind to the original document) retranslated the document back into English. Any differences identified were reviewed with Emirati and Western researchers and modified to minimise semantic differences.

A pilot study was conducted in which ten Emirati women, who had just given birth in the government maternity Corniche Hospital (Abu Dhabi), were recruited. Results from this pilot initiated further adaptations to the study designed to account for maternal literacy and the number of visitors in the mother’s hospital room.

All Emirati women who gave birth in the Corniche Hospital over the period of 1st October to the 1st November 2002 were invited to participate in the study. Around 10% of the eligible participants declined to take part in the study, primarily due to ill health or because they were refused permission from their male guardian. An Arabic-speaking female research assistant interviewed mothers during their postpartum stay in the hospital. Additionally, the women’s medical records were reviewed and then they were contacted via mail and/or telephone at three (*n* = 94), six (*n* = 59) and 15 months postpartum (*n* = 52).

Apgar scores were used to provide an assessment of the overall general health and condition of the baby [16]. Apgar scores range from 1 to 10 with above 7 being normal and below three indicating that the infant is in critical condition [16]. Apgar scores also provided a subjective numerical categorisation of each new born with respect to heartbeat, respiratory rate, colour, muscle tone and response to stimuli.

Data were analysed using IBM SPSS Statistics package Version 23. Fisher’s exact test and adjusted odds ratios and their 95% confidence limits were used to assess significant relationships between LBW and a range of explanatory factors.

## Results

The demographics of the participants are shown in Table 1 along with anthropometric measurements of the infants at birth.

**Table 1:** Characteristics of mothers & infants

Participant characteristics			
Maternal			
Age (mean, SD, range)	28.7	5.7	16–46
Age at marriage (mean, SD, range)	20.8	4.5	11–38
Parity (mean, SD, range)	3.4	2.1	1–9
Primiparous (n, %)	29	23	
Education level (n, %)			
None	6	5	
Primary	28	22	
Secondary	62	50	
Diploma/degree	29	23	
Working before birth (n, %)	36	29	
Consanguineous marriage (n, %)	60	48	
Polygamous marriage (n, %)	7	6	
Infant			
Sex (n, %)			
Male	62	49.6	
Female	63	50.4	
Gestation (mean, SD, range) weeks	39.1	2.4	25–44
Birthweight (mean, SD, range) kg	3.2	0.6	0.7–4.4
Length (mean, SD, range) cm	51.5	3.1	41–60
Head circumference (mean, SD, range) cm	34.6	1.7	24–40

At birth, 11 (9%) of the infants were LBW. Table 2 lists the covariates considered to potentially influence infant LBW. The univariate odds ratios indicate the likelihood that the infant is of normal birth weight.

**Table 2:** Factors influencing the likelihood of low birth weight (< 2.5 kg). Numbers (percentage), Fisher’s exact test probability and univariate common odds ratios (95% confidence intervals) are listed. Significant associations are denoted by * and are bolded. The common odds ratio greater than 1.0 indicates an association between that character and birth weight (in the sense that having normal birth weight raises the odds of having that character, relative to having LBW)

Variables	Birth weight less than 2.5 kg				Fisher’s Exact test *p* value	OR	Lower 95% CI	Upper 95% CI
	YES		NO					
	^*^N (11)	%	^*^N (114)	%				
Maternal age					1.00			
< 29	6	8.96	61	91.04		1.00		
≥ 29	5	8.62	53	91.38		1.04	0.30	3.61
Maternal age at marriage					0.210			
< 21	3	5.00	57	95.00		1.00		
≥ 21	8	12.31	57	87.69		0.38	0.09	1.48
Consanguineous marriage								
yes	2	3.39	57	96.61	0.057	1.00		
no	9	13.85	56	86.15		0.22	0.06	1.06
Number of live births					0.758			
< 4	7	9.72	65	90.28		1.00		
≥ 4	4	7.55	49	92.45		1.32	0.37	4.76
Birth mode					0.724			
Vaginal	8	8.42	87	91.58		1.00		
Caesarean	3	10.00	27	90.00		0.83	0.21	3.34
Infant gender								
male	3	4.84	59	95.16	0.205	1.00		
female	8	12.70	55	87.30		0.35	0.09	1.34
Infant sent to NICU					0.000*			
yes	5	45.45	3	54.55		1.00		
no	6	2.63	111	97.37		30.83	5.92	160.60
Education level					0.669			
none or Primary	4	11.76	30	88.24		1.00		
Secondary	4	6.45	58	93.55		0.14	0.03	0.72
Tertiary	3	10.34	26	89.66		0.28	0.06	1.27
Mother currently working					0.728			
yes	7	7.87	82	92.13		1.00		
no	4	11.11	32	88.89		0.68	0.19	2.49
Mother had regular check-ups during pregnancy					1.000			
No	1	6.25	15	93.75		1.00		
yes	10	9.17	99	90.83		0.66	0.08	5.53
Pre-existing maternal anaemia					0.689			
No	9	8.57	96	91.43		1.00		
yes	2	10.00	18	90.00		0.84	0.17	4.23
Anaemic status					0.196			
No	8	7.48	99	92.52		1.00		
yes	3	16.67	15	83.33		0.40	0.09	1.70
Maternal diabetes					0.579			
No	10	8.70	105	91.30		1.00		
yes	1	11.11	8	88.89		0.76	0.09	6.73
Folic acid intake during pregnancy					0.202			
No	2	4.17	46	95.83		1.00		
yes	9	11.70	68	90.67		0.33	0.07	1.59
Iron intake during pregnancy					0.042*			
No	5	20.0	20	80.0		1.00		
yes	6	6.0	94	94.0		3.92	1.09	14.10
Multivitamin and mineral intake during pregnancy					0.11			
No	9	13.04	60	86.96		1.00		
yes	2	3.64	53	96.36		3.98	0.82	19.22
Primiparous					0.716			
yes	3	10.34	26	89.66		1.00		
No	8	8.33	88	91.67		1.27	0.31	5.13
Infant required resuscitation					0.330			
No	5	5.0	95	95.00		1.00		
Yes	2	10.00	18	90.00		0.47	0.09	2.63

Missing data. The following variables had missing data: Maternal diabetes (1 case), Multivitamins and minerals taken during pregnancy (1 case), Infant required resuscitation (5 cases)

No associations were found between birth weight and maternal age, age at marriage, consanguinity, education level, current maternal employment, primiparity, pre-existing anaemia or anaemia in pregnancy, diabetes, folic acid intake or multivitamin intake or infant gender. Iron intake during pregnancy was associated with fewer LBW infants (Fisher’s exact test *p* = 0.042). Mothers taking iron supplements were 3.9 times more likely to have normal weight babies than those not taking iron supplements.

LBW infants were significantly more likely to require treatment in the neonatal intensive care unit (NICU) [OR = 30.83, *p* = 0.00]. Seven of the infants (6%) were born preterm and as expected were more likely to be admitted to the NICU. Four of the infants were small for gestational age suggesting that, if their recorded gestational ages were accurate, they had suffered IUGR.

All but three of the 11 LBW infants weighed more than 2 kg. The lightest infant was born at 26 weeks gestation and weighed 710 g, whilst another was born at 25 weeks gestation, weighing 780 g. The third infant weighed 1.49 kg and suffered cardiac issues but remained in the study for the duration. Eight of the infants in the study were admitted to the NICU immediately after birth. The reasons for admission varied and included: preterm/very low birth weight; pre-term/intrauterine growth restriction; ileal atresia; tachypnoea; and congenital myopathy. No relationships between NICU admittance and consanguinity, maternal age, or education level, regular check-ups during the pregnancy, or maternal desire for the pregnancy were found.

The Apgar scores taken at one and 5 min after birth were slightly higher for the boys than for the girls at 1 min but this was not statistically significant. No significant relationships were found between length of gestation period and birth weight with Apgar scores. No infant received a critical score at 5 min after birth.

Data relating to the initiation and duration of breastfeeding and consumption of additional liquids and foods during infancy in this cohort have been extensively reported in a previous publication [17]. Exclusive breastfeeding rates were low and associated with perceptions of insufficient milk supply, infant hunger, and maternal employment. Early introduction of supplementary food and drinks was common, some being ritualistic in nature. Maternal employment and pre-lacteal feeds were significantly related to the early introduction of supplementary foods. However, 50% of the mothers interviewed on follow up at 15 months were still giving breast milk.

At 15 months of age most of the infants were taking meals with the rest of the family, with only five being fed separately. All the infants consumed a varied diet by 14 or 15 months, eating the same food as the rest of the family at least some of the time. The most commonly consumed foods were: rice, apples, banana, mango, kiwifruit, potato, squash, carrots, beans, meat, fish, confectionery, eggs, biscuits, bread, yogurt, cheese. All the infants, with just one exception, consumed French fries. Twenty-five (48%) of the infants were reported as frequently eating French fries, which were also popular as a snack between meals. Other popular snack choices included: biscuits, confectionery, yogurt and fruit. The infants consumed a range of beverages; water and pure fruit juices being the most popular. Five infants (10%) had consumed tea; four were given coffee (8%), while three (6%) had been given carbonated soft drinks. Many of the participants expressed concern that their baby was not eating enough (*n* = 24, 46%), but only 5 (10%) had concerns regarding infant growth.

One of the most disturbing findings was that only 5.3% (*n* = 5) of mothers reported that their infants were placed in a secure car seat when travelling during the first 3 months of life. Most infants were held in the arms of an adult in either the front or back seats (Table 3).

**Table 3:** Infant transportation by car in first 3 months of life

Infant travels by car:-	Frequency	Percent
In an adult’s arms in the front seat	70	74.5
In an adult’s arms in the back seat	17	18.1
In a loose infant car seat	1	1.1
In a secured infant car seat	5	5.3
Other	1	1.1
**Total**	**94**	**100.0**

Although the infants did have some health problems, the mothers were apparently reluctant to report their infants as unhealthy, as the lowest maternal perception of infant health report by a mother was that their infant’s health was average as shown in Table 4 below:

**Table 4:** Mothers’ interpretation of their infant’s health

Infant health	Infant age (months)					
	3 (*n* = 93)		6 (*n* = 59)		14–15 (*n* = 51)	
Excellent	13	14.0%	4	6.8%	4	7.8%
Good	19	20.4%	14	23.7%	9	17.6%
Average	61	65.6%	41	69.5%	38	74.5%

It is interesting that over the study period only 7–14% rated their infant’s health as excellent. Table 5 below shows infant health issues by maternal report during each period of the study which included a variety of ailments with fevers, colds/flu and coughs being most commonly reported.

**Table 5:** Frequency of health problems suffered by infants as reported by their mothers

	Infant Age (months)					
Illness	3 (*n* = 81)		6 (*n* = 47)		14–15 (*n* = 40)	
Fever	23	28.4%	21	44.7%	20	50.0%
Cough	35	43.2%	27	57.4%	25	62.5%
Cold/flu	36	44.4%	19	40.4%	20	50.0%
Vomiting	11	13.6%	9	19.1%	10	25.0%
Diarrhoea	9	11.1%	11	23.4%	11	27.5%
Breathing problems	12	14.8%	13	27.7%	6	15.0%
Ear infection	3	3.7%	12	25.5%	9	22.5%
Rash/skin infection	17	21.0%	8	17.0%	5	12.5%

Chronic infant health issues reported included: asthma (*n* = 7, 6%), eczema (*n* = 1, 1%), food allergies (*n* = 1, 1%), heart problems (*n* = 1, 1%) congenital myopathy (*n* = 1, 1%) and eye problems (*n* = 1, 1%).

At 3 months after birth, only three (3%) of the infants had not received all the recommended vaccinations, due to infant illness and lack of transportation. Six (7%) infants had not had a health check. Sixty eight of the infants (81%) had between one and four check-ups during the first 3 months of life, and ten infants (12%) had five or more. The reasons given for not taking infants for health check-ups included: baby was not ill, mother was too busy, mother was ill, lack of transport, husband would not take mother to clinic, lack of knowledge on how to make an appointment.

Data collected at 6 months after birth showed that all but one of the infants were now up to date with their vaccinations; the exception being due to problems with transportation and ill health.

By 14–15 months, all the infants (*n* = 52) had received medical check-ups. Thirty-two (62%) of the infants had received between four and seven check-ups while six (12%) had received more than eight. Reasons given for not having regular health checks for the infant included: infant was not ill (*n* = 12, 23%), mother was too busy (*n* = 2, 4%), lack of transportation (*n* = 2, 4%), and one woman said that her husband would not take her. All infants had received all the recommended vaccinations, although two were behind schedule.

As shown in Table 6, most of the women took the infants to government clinics and hospitals for medical treatment, although a substantial number used private facilities. Many used government clinics or hospitals to have their infant vaccinated and private facilities when the infant was ill.

**Table 6:** Utilisation of medical facilities for care of infants

	Infant age (months)					
Health care provider	3 (*n* = 92)		6 (*n* = 59)		14–15 (*n* = 51)	
Government hospital	26	28.3%	13	22.0%	19	37.3%
Government clinic	34	37.0%	30	50.8%	24	47.1%
Private hospital	17	18.5%	9	15.3%	13	25.5%
Private clinic	31	33.7%	20	33.9%	23	45.1%
Pharmacy	1	1.1%	0	0.0%	1	2.0%
Traditional healer	3	3.3%	0	0.0%	0	0.0%

Most participants relied on doctors to prescribe medication when the infant was ill. Very few of the women went directly to the pharmacist or shop to purchase medicine, as shown in Table 7. Interestingly, several of the participants used medicines supplied by traditional healers to treat their infants. Traditional medicines were often used first, and if they were not effective, then the infant was taken to a hospital or clinic.

**Table 7:** Sources of infant medications

	Infant age (months)					
Medication prescribed by:	3 (*n* = 65)		6 (*n* = 52)		14–15 (*n* = 40)	
Doctor	61	93.8%	28	53.8%	37	92.5%
Pharmacist	2	3.1%	4	7.7%	1	2.5%
Traditional healer	4	6.2%	1	1.9%	1	2.5%
Purchased in shop	1	1.5%	0	0.0%	0	0.0%

At 3 months after birth the respondents (*n* = 94) were asked about information and advice they had received regarding breastfeeding while in the hospital. Forty-eight (51%) of the women had received information regarding breastfeeding from hospital staff. The staff in the hospital had helped 63 (68%) of the participants to breastfeed during their stay in the hospital. However only 20 (22%) of the women reported receiving a phone number from the hospital staff to call midwives for help if they experienced any subsequent problems with breastfeeding. Only one mother who had decided to bottle feed before the birth was given advice on how to make bottles and given a gift pack containing samples of infant formula.

At 6 months after the birth, participants (*n* = 58) were asked where they obtained information relating to the introduction of complementary foods. Most women found information through books and magazines (*n* = 25, 43%), government health establishments (*n* = 23, 40%), private health establishments (*n* = 14, 24%), and family, friends or personal experience (*n* = 16, 28%) while four women obtained information from the television and internet sources (7%).

The participants were also asked if they had received information relating to Sudden Infant Death Syndrome (SIDS). Only nine of the 58 women had heard of SIDS and six of these had found out about it from television, two from family and friends and one from a doctor.

## Discussion

The population of Abu Dhabi, the wealthiest of the seven emirates of the UAE, has experienced an exceptionally rapid transition from a subsistence existence to one of wealth and privilege within the space of 40 years. One would expect that infant health would improve during this transition due to access to better health resources. Indeed, this paper confirms that LBW, an important aspect of infant health at birth, has improved in Abu Dhabi and is similar to that of developed countries.

LBW is a risk factor for numerous infant health issues, both acute and chronic. This study indicated an incidence of 8.8% of LBW; an improvement from the figures shown in the UAE Family Health Survey based on data from all the emirates, which found an incidence of 15% in 1995 [18]. These figures are comparable to other Arab countries such as; Jordan, 13% and Oman,12% (2007–2011 data) [9]. The rate of infant LBW in developed countries such as the UK and US is approximately 8% with a world average of 15% [9]. This decrease in the incidence of LBW is encouraging as it indicates the effectiveness of public health policy in the improvement of infant health in the UAE.

Between 1992 and 1999, the UAE neonatal mortality rate was reported as 6.9 per 1000 live births, with preterm birth complications and lethal malformations accounting for 77% of all such deaths [19, 20].

Consanguineous marriages are common in the UAE and marriages between first cousins occur frequently [21]. It is estimated that there are at least 213 genetic disorders and congenital abnormalities in the UAE population, many of which are likely a result of the practice of consanguineous marriage [22]. Between 1995 and 1997, the incidence of major congenital abnormalities was reported to be 23/1000 [19]. While our study did not address major congenital abnormalities, and mothers of such infants were unlikely to have participated in the study, levels of consanguineous marriage were high. Almost half (48%) of the mothers were related to their husband. This is very similar to previous findings; the UAE Family Health Survey conducted in 1995 found 40% of UAE women were blood relatives to their husband [18]. Bener et al. [23] found 50.5% of marriages to be consanguineous in a sample of 2200 women. However, in the present study no relationship was found between babies born to mothers in a consanguineous relationship and LBW or admittance to the NICU. While this could reflect increased awareness and pre-marital screening for genetic incompatibilities, it could also result from selection bias, with mothers of VLBW infants or infants with congenital abnormalities being less likely to participate in the study.

The number of infants born to adolescent women is declining in the UAE. Within this sample five women (4%) were below the age of 20 years. Green and Smith [24] found that the mean age at which UAE mothers gave birth to their first child increased across three generations from 15.9 to 20.9 years. This decrease in adolescent births is an important change given their associated health problems. Shawky and Milaat [25] reported that Saudi Arabian women who gave birth before age 16 had double the risk of developing chronic ill health and experiencing miscarriages, stillbirths and infant deaths throughout their entire childbearing years.

Anaemia is viewed as a serious health problem in the Eastern Mediterranean region and WHO indicators for reproductive health show that 40.9% of women screened for anaemia during pregnancy had haemoglobin concentrations below 110 g/l [26]. In the UAE, the prevalence of anaemia is not well documented. WHO figures show that 22–62% of pregnant women in the UAE were anaemic in 1995, but this had decreased to 14% in 2002 [27]. Fareh et al. conducted a study in Al Ain examining the obstetric impact of anaemia during pregnancy and recorded that 13.3% of pregnant women attending Al Ain hospital during the study period were anaemic. However, their study found no significant adverse effects of low iron on mothers or infants, likely due to good standards of ante-natal care [28]. There are several characteristics of the UAE diet which may inhibit iron absorption including a high consumption of tea, which contains tannins, and large quantities of unleavened bread containing phytates [27]. It is common for women in the UAE to have many children and therefore to be in a constant cycle of pregnancy and lactation, which does not allow replenishment of iron stores, resulting in iron deficiency anaemia [24, 28, 29].

Iron supplementation is common in the UAE and supplements are routinely prescribed at maternity clinics, although this is only effective if women attend antenatal clinics early in pregnancy. The efficacy of supplementing women with iron pills during pregnancy to prevent and treat anaemia is well documented, although in developing countries, consideration also has to be given to the possible presence of other micro-nutrient deficiencies [30, 31]. Results from the current study suggest that iron supplementation had a positive impact on infant birth weight. However, further investigation is needed to confirm this result and a larger sample size may prove to be more informative.

The prevalence of breastfeeding and the introduction of complementary foods in this population has been previously reported by Gardner et al. [17]. Although 50% of the infants were still receiving some breastmilk at 15 months they were also consuming a diet high in fatty and sugary foods. This may be reflected in the high levels of overweight (21.5%) and obesity (13.7%) reported in children aged 5–17 in the UAE [32].

The infants in this study suffered from the usual array of common childhood ailments. In addition, chronic infant health issues reported included: asthma, eczema, food allergies, heart problems, congenital myopathy and eye problems. Breathing difficulties and rashes were common suggesting that allergies may be common amongst this cohort. The prevalence of asthma and eczema in children in the Emirates has been reported as 13% and 11% respectively [33]. A more recent study in Al Ain found that 8% of school aged children suffered from food allergies [34]. These rates are similar to those reported in developed countries, and more research is needed on the causes and prevention of these allergies in the UAE [35].

Several of the mothers reported that their child had more serious or chronic conditions but these were relatively rare. The nature of the health services in Abu Dhabi offers a range of choices in health care or provision of medication. This was particularly the case in relation to medication with six of the participants relying on traditional healers to provide remedies for the infants. Depending on the composition of the remedy this may be cause for concern as some traditional herbal remedies have been associated with toxins or contamination, and may pose a threat to health [36].

Mortality and injury resulting from road traffic accidents are common in the UAE [37, 38]. Although this study found that only 5.3% of infants were secured in car seats, the reluctance to use car seats for infants has recently been improved through public awareness campaigns and legislation. The Abu Dhabi government passed legislation in 2011 to make it mandatory to place infants in car seats [39], although non-compliance remains a problem [38] with a recent study reporting that 44% of respondents claimed to never use a secure car seat for their children between birth and 23 months [40].

## Study limitations

There are several limitations to this study that should be noted. These include subject attrition and low response rates on follow up, largely due to the mobility of this population, with many of the new mothers moving between extended family residences. This population was relatively healthy as mothers with ill or low birth weight infants may have declined to participate in the study. In addition, mothers in this culture may not be willing to admit to poor health in their children and health status may therefore be subjective. The use of the Apgar scores as a proxy of overall general health of new-borns has limitations. The exploratory descriptive nature of this study depicts a unique set of circumstances documented at a single point in time in Abu Dhabi, a rapidly developing city. This, combined with the relatively small number of subjects, serves to limit any claims this study might make about representativeness of the entire population.

## Conclusions

The health of infants born to the mothers in this UAE sample from Abu Dhabi showed marked improvement over previous studies. Although consanguinity among parents was high, no evidence for negative impacts on birth weight or prematurity was found. The proportion of LBW infants was decreasing and continuing improvements in health care in the UAE are having a positive impact on infant health. To further improve infant health outcomes for mothers and infants in the United Arab Emirates, more research and the implementation of health education programmes would be beneficial.

## Abbreviations

ELBW: Extremely low birthweight
IUGR: Intrauterine growth restriction
LBW: Low birthweight
NICU: Neonatal intensive care unit
SGA: Small for gestational age
VLBW: Very low birth weight
